# Editorial: New ideas in language sciences: language acquisition

**DOI:** 10.3389/fpsyg.2023.1250307

**Published:** 2023-07-27

**Authors:** Itamar Lerner, Pedro Guijarro-Fuentes, Roberto Filippi, Fangfang Li, John W. Schwieter

**Affiliations:** ^1^Department of Psychology, The University of Texas at San Antonio, San Antonio, TX, United States; ^2^Department of Spanish, Modern and Classic Philology, University of the Balearic Islands, Palma, Spain; ^3^UCL–Institute of Education, University College London, London, United Kingdom; ^4^Multilanguage and Cognition Lab, UCL–Institute of Education, London, United Kingdom; ^5^Department of Psychology, University of Lethbridge, Lethbridge, AB, Canada; ^6^Language Acquisition, Multilingualism, and Cognition Lab/Bilingualism Matters, Wilfrid Laurier University, Waterloo, ON, Canada; ^7^Department of Linguistics and Languages, McMaster University, Hamilton, ON, Canada

**Keywords:** language, language acquisition, L2, L3, bilingual

Language acquisition has now become a fast-growing field, owing to significant advancements in recent years in both empirical studies of humans and animals and theoretical models of language development. Particularly, meteoric advancements in generative artificial intelligence such as *chatGPT*, which demonstrates an almost human-like ability for comprehending and producing discourse, highlight the importance of understanding the way real brains acquire and exhibit linguistic abilities. In this Research Topic, we present a collection of experimental studies and reviews that shed light on recent progress made in language acquisition in humans. Articles include highly contemporary topics such as second (L2) and third (L3) language acquisition, production vs. comprehension, statistical learning in language, and others.

Jiang and Zhang examined the interface hypothesis (Sorace, 2011, 2012), which argues that the syntax–pragmatics interface poses the biggest difficulty and learning delays in attaining high-level L2 acquisition. They focused on the way English native speakers and Chinese learners of English handle existential constructions, which crucially require a mapping between syntactic forms and pragmatic functions. Results showed that before reaching an advanced learning stage, Chinese learners overproduced and exhibited an inappropriate preference pattern for existential constructions, stemming from various influences of their first language (L1). The results, therefore, supported the interface hypothesis and can be used for developing practical teaching strategies when acquiring an L2.

Chen and van de Weijer also investigated L2 acquisition but from the perspective of acoustic production. Particularly, they investigated whether producing similar-sounding consonants, in this case, post-alveolar fricatives, differs between Chinese monolingual and bilingual individuals when speaking in English, a less familiar language. It was found that in some cases, the accentedness ratings and acoustic traits of the produced consonants provided contrasting results. For example, the production of/∫/(“sh”) by the bilinguals was judged to have no accent despite being acoustically more distant from its native version compared to monolinguals. This finding suggests that L1–L2 dissimilarity, assumed to predict L2 acquisition success, must be considered not only from the learners' perspective but from the native speakers' perspective as well.

In a review of L2 literature, Lin et al. assessed L2 pronunciation among children in bilingual education programs. Using a narrative literature review approach, the authors put forth a conceptual model of L2 speech in communicative situations that unraveled L2 learner–interlocutor interactions across three layers: the sociopsychological, acquisitional, and productive-perceptual layers. In particular, the productive-perceptual layer (c.f., “speech circuit,” De Saussure, 1959) emphasizes the role of L2 learners' pronunciation in L1 listeners' perceptions, which, in turn, is also affected by sociopsychological and acquisitional factors from the other layers. The model offers a foundational tool on which researchers, practitioners, and educators can build new ideas and situate novel research agendas.

In another systematic review, Planckaert et al. explored the evidence for potential cognitive advantages associated with bilingualism, particularly in the areas of inhibition and switching as they relate to executive functioning. The authors show that the current literature pointing to a bilingual advantage has been mixed, with somewhat more consistent findings in young children and aging adults and less so for older children. Therefore, the critical period for cognitive development and cognitive decline appears to be particularly sensitive to bilingualism.

Liu provides a final investigation of non-native language acquisition, performing a bibliometric study of how scientific research on L3 acquisition—an emerging topic of inquiry in its own right (Leung, 2007)—has developed over the last decades. By looking at 425 publications from the Scopus database, it was found that this research began to spark in 2015 after three decades of development. Liu identifies what journals are keen to publish L3 research, who are the leading researchers in the field, and which countries contribute most to the discipline. Overall, this bibliometric study provides directions into the evolving trends of L3 acquisition studies, which can help scholars identify new research trends and gaps in the field.

Moving on to other areas of language acquisition, Ji et al. examined the effect of word order on children's production and comprehension of sentences in their native language. Mandarin-speaking children aged 3–6 years were tested on both comprehension and production of non-canonical word order compared to standard word order. Non-canonical word order was found to be more difficult for children under the age of 6 years, particularly in sentences with passive structures. Comparing two prominent theoretical frameworks in the field, the authors show that the results are slightly more compatible with the usage-based approach, which argues that syntactic constructions gradually develop with exposure to input, and less so with the maturational account, which proposes that some underlying structures are innate and become mature with age.

The issue of testing linguistic skills is central to Liao et al.'s mini-review exploring the current status of communicative language testing (CLT), a decades-old methodology that focuses on the ability to communicate in a newly learned language in authentic contexts. The authors describe the three dominant approaches in CLT, namely theory-based, real-life, and integrated approaches (Harding, 2014), as well as the challenges that CLT faces, including operationalizing any model into specific test designs and the tension between the validity and reliability of tests.

Finally, in another focused review, Roembke et al. discuss the evidence supporting cross-situational word learning (CSWL), a phenomenon in which language learners acquire word meanings by tracking statistical co-occurrences between words and objects across multiple situations. The authors show that while CSWL has been demonstrated across ages, the long-term durability of the acquired word representations is often unclear and the simplicity of the statistical relationships in these studies raises questions about the applicability of CSWL to more complex language learning scenarios.

Together, the studies presented in this Research Topic demonstrate the current breadth of research conducted in the language acquisition domain. They urge us to consider not only obvious modulators of performance such as stimuli characteristics but also non-trivial influences emerging from factors such as contextual and sociopsychological aspects of learning environments. Finally, all studies emphasize the importance of future research in clarifying yet unanswered questions in the field, of which there are many.

## Author contributions

IL wrote the first draft of the editorial. PG-F, RF, FL, and JS reviewed and edited the editorial. All authors contributed to the article and approved the submitted version.
